# A Case of Transient Gestational Hyperthyroidism Complicated With Acute Liver Injury Successfully Treated With Plasma Exchange

**DOI:** 10.7759/cureus.29442

**Published:** 2022-09-22

**Authors:** Na Zhou, Feng Zhang, Cyril Kuriakose, Dwayne Gordon, Amay Parikh

**Affiliations:** 1 Internal Medicine, AdventHealth Orlando, Orlando, USA; 2 Biochemistry, Duke University, Duram, USA; 3 Nephrology, AdventHealth Orlando, Orlando , USA; 4 Intensive Care Unit, AdventHealth Orlando, Orlando, USA

**Keywords:** thyrotoxicosis, pregnancy, acute liver injury, transient gestational hyperthyroidism, plasma exchange

## Abstract

Therapeutic Plasma Exchange (TPE) in pregnancy is rare due to the uncertainty of efficiency and safety in clinical practice. It is experience-based on extrapolation of efficacy and safety in non-pregnant populations than evidence-based. We reported a case of severe refractory hyperemesis gravidarum secondary to transient gestational hyperthyroidism in a first-trimester pregnancy, which was complicated by acute hepatic injury during the clinical course and successfully managed with TPE. Both the clinical picture and objective index were improved dramatically after plasma exchanges. Three sessions of plasma exchange provided a 61% decrease in serum FT4 (free thyroxine) concentration (4.34 ng/dL to 1.71 ng/dL) and 89% decrease in alanine aminotransferase (ALT) (647 units/L to 69 units/L). The patient's symptoms improved significantly after TPE. In two weeks follow-up visit, her thyroid function was in the recommended range of 1st-trimester pregnancy (1.06 ng/dL) and her liver function was normalized (ALT 15 units/L, aspartate aminotransferase {AST} 11 units/L). In conclusion, plasma exchange may be used as an alternative therapeutic option in pregnancy to manage transient hyperthyroidism who failed or was unable to tolerate or have contradictions to antithyroid medications and thyroidectomy. Our case provides evidence of TPE in the treatment of thyrotoxicosis in pregnancy.

## Introduction

Gestational transient thyrotoxicosis (GTT) is one of the most common causes of hyperthyroidism in pregnancy which results from the stimulatory action of human chorionic gonadotropin (HCG) on the TSH receptor. If left untreated, severe cases are associated with increased risks of adverse maternal and fetal complications. Therapeutic options are limited due to the potential adverse fetal effects of the available treatment. Propylthiouracil (PTU) is the preferred thioamide in the first trimester due to fewer birth defects. Radioiodine is contraindicated and surgery, if indicated, should be performed during the second trimester. In other words, PTU combined with beta-blocker is the main available therapeutic option during the first trimester based on current guidelines [1,2]. We encountered a case of severe refractory hyperemesis gravidarum likely due to thyrotoxicosis in the first trimester, she was not able to tolerate any oral intake including antithyroid medication even with multiple antiemetics and steroids. This case is also complicated with acute liver injury due to uncontrolled hyperthyroidism. TPE was employed with subsequent improvement of both clinical symptoms and objective index.

## Case presentation

**Figure 1:**
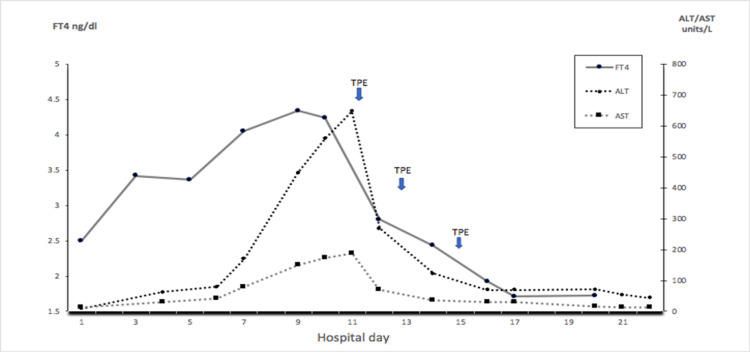
FT4 and liver enzymes before and after TPE FT4: Free thyroxine; TPE: Therapeutic plasma exchange

A 31-year-old woman gravida 3 and para 2 presented with progressively worsening nausea and vomiting in the past two weeks. The pregnancy was confirmed with a positive serum beta-human chorionic gonadotropin (b-HCG) and ultrasonography (US) of the pelvis consistent with single living intrauterine gestation of eight weeks five days. Her medical history includes hyperthyroidism in both of her prior pregnancies with the second one being treated with PTU. Physical exam with no thyromegaly and ophthalmopathy, no tremor. Vitals with borderline tachycardia, otherwise were within normal limits. Laboratory investigation revealed beta-HCG 12000 IU, free thyroxine (FT4) 2.5 ng/dl (0.58-1.64 ng/dl), thyroid stimulating hormone (TSH) 0.008 mIU/L (0.4-4.5 mIU/L), thyroid peroxidase antibody (TPO) (-), thyroid-stimulating immunoglobulin (TSIG) (-) urine ketone 4+. Normal complete blood count (CBC), liver and renal function, mild hyponatremia 134 mmol/L (135-145 mmol/L) and hypokalemia 3.2 mmol/L (3.5-5.0 mmol/L). After admission, the patient received intravenous (IV) hydration including vitamin B, antiemetics, and PTU. Unfortunately, in the subsequent days, the patient failed multiple antiemetics and methyl prednisone, nausea and vomiting continued to get worse, she was not able to tolerate any oral intake and elevated liver enzymes were noted on day 5 and continued to trend up with maximum alanine aminotransferase (ALT) 647 units/L (4-51 units/L) and aspartate aminotransferase (AST) 186 units/L (5-46 units/L) on day 10 (Figure 1 and Table 1). Liver injury panels including viral hepatitis, smooth muscle antibody, mitochondrial antibody, liver kidney microsomal antibody, Alpha-1 antitrypsin, Fe/TIBC (total iron binding capacity) ratio, and serum ceruloplasmin were all inconclusive. PTU was contradicted since her ALT/AST level was above three times the upper limit of normal.

**Table 1:** Hormone and liver enzyme levels before and after TPE Normal values: FT4 0.58-1.64 ng/dL; TSH 0.4-4.5 mIU/L; ALT 4-51 units/L; AST 5-46 units/L. FT4: Free thyroxine; TSH: Thyroid-stimulating hormone; ALT: Alanine aminotransferase; AST: Aspartate aminotransferase

Hormone/Enzymes	On admission	Before TPE	After TPE		
			1st	2nd	3rd
FT4, ng/dL	2.5	4.34	2.8	2.23	1.71
TSH, IU/mL	0.008	<0.005			
ALT, units/L	9	647	260	123	69
AST, units/L	12	186	72	35	29

Due to exhaustion of conservative medical management and worsening clinical scenario, TPE was introduced and carried out via a non-tunnel catheter once every other day (TPE parameters listed in Table 1). Three sessions of plasma exchange provided a 61% decrease (4.34 ng/dL to 1.71 ng/dL) in serum FT4 concentration and 89% decrease in ALT (647 units/L to 69 units/L). The patient’s nausea and vomiting were significantly improved, and FT4 and liver enzymes trended down nicely. At the two weeks post-TPE follow-up, her thyroid function was in the recommended range of first-trimester pregnancy (1.06 ng/dL) and her liver function was normalized (ALT 15 units/L, AST 11 units/L).

**Table 2:** TPE Parameters TPE: Therapeutic plasma exchange; FFP: Fresh frozen plasma

Device	Spectra Aptia Apheresis system
Manufacturer	TERUMOBCT (Terumo Blood and Cell Technologies, USA)
Anticoagulant (AC)	ACD (Anticoagulant Citrate Dextrose Solution Formula A)
AC ratio	10:1
Plasma volume treated	3.5 L
Replacement fluid	Albumin: FFP=1:1
Number and frequency	Three procedures, every other day

## Discussion

The patient presented with elevated thyroid function tests occurring in the context of hyperemesis, given the absence of pre-pregnancy hyperthyroidism, the lack of stigmata of hyperthyroidism, and the absence of anti-TSH receptor antibodies, a diagnosis of GTT was made. Although most cases of GTT are mild and not associated with significant obstetrical complications and adverse neonatal outcomes, children born to mothers experiencing GTT complicated by severe hyperemesis and weight loss of > 5% of their pre-pregnancy weight have been reported to have significantly lower birth weight as compared to gestational-age matched infants born to unaffected mothers [3,4] and may need anti-thyroid treatment.

Acute liver injury is not uncommon in GTT. The most common liver laboratory abnormalities are mild aminotransferase elevation which rarely goes more than 200 IU/L and the severity of nausea and vomiting likely correlates with the degree of liver enzyme elevation [5-6]. Our patient presented with hyperemesis gravidarum, did not respond to supportive management, and her case was complicated with acute liver injury with ALT> 600 which makes PTU contradicted. The patient’s symptoms and liver function continued to deteriorate, after extensive multidisciplinary team discussion and full counseling of the patient and family about the risks and benefits, we decided to implement TPE treatment.

The use of TPE in pregnancy is very limited due to a lack of evidence-based safety and efficacy in clinical practice. The current application of TPE in pregnancy is largely empiric and based on experience in non-pregnant populations and individual case reports in the absence of large-scale and high-quality studies. The main concern of TPE during pregnancy is the fetal compromise from potential safety issues including hypotension, allergic reaction from plasma replacement and vascular access associated infection, etc. However, the safety issues of TPE in pregnancy seem to have a similar profile compared with non-pregnancy based on current data although it’s somewhat limited [7-9].

Here are some other considerations when implementing TPE in pregnancy: (1) Maternal plasma volume started to increase from the end of the first trimester, can increase by 25-30% at the end of the second trimester, and 40-50% near term with the steepest rate of increase in the 2nd trimester. Nadler's equation does not count for this change. We must consider this dramatic increase and adjust plasma volume based on pregnancy duration to ensure effective exchange. It’s not applied in our case due to the patient being in the 1st trimester, (2) Albumin is the most commonly used replacement fluid with the advance of less transfusion reaction and viral transmission. But in certain diseases such as TTP, coagulopathy, and bleeding plasma is preferred to replenish deficient plasma protein or coagulation factors. we elected to modify the exchange fluid from solely 5% albumin to a 1:1 ratio of 5% albumin to fresh frozen plasma since it has been proposed that plasma has the advantage of providing thyroid bounding proteins in addition to supplementing coagulation factors, (3) We recommended early involvement of maternal-fetal medicine to ensure maternal and fetal safety. In our case, daily doppler measurement and cardiotocography were conducted with no evidence of fetal compromise. More frequent monitoring was performed in some studies; however, no advent effect was reported on fetal doppler or cardiotocography suggesting fetal compromise when material intravascular volume and blood pressure are maintained. Therefore, the benefit of more frequent monitoring is not established, (4) During the procedure, the patient in the 2nd and 3rd trimester should be positioned slightly in the left lateral position to minimize the compression of inferior vena cava (IVC) from the enlarging uterus, (5) Last but not the least, TPE is a dramatic procedure and has risks, enough psychological and emotional support should be provided to both patients and families.

It is important to mention that all available knowledge regarding TPE during pregnancy is limited to case reports and a few cohort studies. We must fully weigh the pros and cons and extensively counsel patients and their families about the risks and benefits before implementing TPE. Given the little evidence available on TPE in pregnancy, it is recommendable to monitor the indications, safety, and efficacy closely and collect these data in a joint effort. Hopefully, with more data and cases available in the future, we can establish a pregnancy-specific plasma exchange protocol considering pathophysiological changes in pregnancy and promote the efficacy and safety of TPE in pregnancy.

## Conclusions

Therapeutic plasma exchange may be used as an alternative therapeutic option in pregnancy in the management of transient hyperthyroidism who failed or was not able to tolerate or have contradictions to antithyroid. Our case provided new evidence for TPE in treatment of thyrotoxicosis in pregnancy. 
